# The Principles and Practice of Surgery

**Published:** 1861-05

**Authors:** 


ART. IX.—*The Principles and Practice of Surgery.* By WILLIAM PIRRIE, F.R.S.E. Second edition. 8vo., pp. 878. John Churchill: London, 1860.

WE are glad to see that the work of the distinguished Aberdeen Professor has reached a second edition, a distinction justly due to its merits; for we have always regarded it as the best outline of surgery in the English language. The first edition was issued in 1852, and was soon after reproduced in this city, under the supervision of Dr. John Neill, at the time Demonstrator of Anatomy in the University of Pennsylvania. By economy of space, and the employment of smaller type, Mr. Pirrie has been enabled to introduce into the volume, without at all increasing its bulk, an amount of new matter equal to nearly one-third of the original. This addition greatly enhances the value of the book, which will thus be still more sought after by the student and young practitioner for whose benefit it has been chiefly prepared. The style of Mr. Pirrie is remarkable for its clearness, and the manner in which he has treated the subject shows him to be a perfect master of it. He occasionally refers to American writers, and we notice one or two paragraphs where the printer has omitted the inverted comma where the author evidently intended it to be used. Such omissions are always exceedingly vexatious to an author, inasmuch as they tend to place him in a false position before the public. The volume is beautifully printed, and the engravings, which are quite numerous, are executed in the highest style of the art. We may add that Mr. Pirrie has not omitted to notice any subject properly appertaining to surgery, thus imparting a degree of completeness to his work, which some of his British brethren might advantageously imitate.

We are not a little surprised that so excellent a surgeon as Mr. Pirrie should still advocate so painful and barbarous a proceeding as the entire removal of the nail in "ingrowing" of this structure. "The proper remedy for this painful affection," he remarks, "is removal of the nail, and afterward treating the ulcer according to the usual rules of practice. To remove the nail, the surgeon firmly grasps the toe with his left hand, passes one blade of a strong-pointed pair of scissors beneath the nail up to its roots, then cuts it through its entire length, and twists off first one half and then the other." Surely the *proper* way is not to do this, but to remove the offending *edge* of the nail on each side, in its entire length, from the matrix forward, the knife being used instead of the scissors.

